# YOLO-FLBM: a lightweight and high-performance model for tomato ripeness detection in complex greenhouse environments

**DOI:** 10.3389/fpls.2026.1824558

**Published:** 2026-06-10

**Authors:** Yuting Su, Peijun Zhang, Huicheng Li, Feng Kang, Pushi Zhao, Lijin Wang

**Affiliations:** 1 College of Computer and Information Sciences, Fujian Agriculture and Forestry University, Fuzhou, China; 2 Key Laboratory of Smart Agriculture and Forestry, Fujian Province University, Fuzhou, China; 3 Engineering Research Center of Smart Sensing and Agricultural Chip Technology, Fujian Province University, Fujian Province University, Fuzhou, China

**Keywords:** complex field environment, lightweight model, ripeness detection, tomato object detection, YOLOv8s

## Abstract

Real-time detection of tomato ripeness in complex greenhouse environments presents a significant dual challenge: the interference caused by foliage occlusion and fruit overlapping demands high detection accuracy, while the limited computational resources of harvesting robots necessitate model lightweighting. To address this, we propose YOLO-FLBM, a lightweight, high-performance model based on the enhanced YOLOv8s architecture. First, the backbone network was reconstructed using FasterNet to minimize redundancy, establishing a streamlined foundation for edge deployment. Second, an innovative neck architecture, designated as the LB Neck, was constructed by integrating the C2f-LS module with the BiFPN structure. Crucially, a novel Multi-scale Coordinate Dynamic Attention (MCDA) mechanism was developed. By integrating hybrid perception pooling with full-rank kernel generation, MCDA dynamically captures spatial dependencies to resolve occlusion issues. Experimental results on a custom tomato dataset demonstrated that YOLO-FLBM achieved comprehensive performance enhancements: precision, recall, mAP@50, and mAP@50–95 reached 95.2%, 91.9%, 97.4%, and 78.9%, respectively, representing improvements of 3.7%, 2.5%, 1.9%, and 1.7% over the baseline model. Meanwhile, the model’s parameter count was reduced to 3.743 M, a substantial 61.9% reduction compared to the original model. These results confirm the model’s efficiency and accuracy, offering a valuable reference for automated tomato harvesting robots.

## 1 Introduction

Tomato (Solanum lycopersicum) is a representative fruit crop with significant economic value in global facility agriculture. It is widely cultivated due to its high nutritional content and extensive consumer demand (Kimura and Sinha, 2008; Tieman et al., 2017). However, the tomato harvesting process still heavily relies on manual operations, especially in facility agriculture environments such as greenhouses, where the fruit distribution is dense, there are significant differences in maturity, leaf shading, and overlapping of fruits are common, making the manual identification and harvesting process highly labor-intensive and time-consuming. With the aging of agricultural labor force, seasonal labor shortage, and the continuous increase in labor costs, the traditional manual harvesting model has become difficult to meet the requirements of large-scale, standardized, and high-efficiency production (Sparrow and Howard, 2021). Therefore, developing automated harvesting technology for greenhouse environments not only helps to improve tomato harvesting efficiency and operational stability but also can reduce long-term labor costs, alleviate seasonal labor pressure, and thereby enhance the economic sustainability of greenhouse tomato production. As a key prerequisite for autonomous harvesting robots to achieve precise operations, quickly and accurately identifying tomato fruits at different maturity stages has become a core issue in current intelligent harvesting systems (Xiao et al., 2023; Huang et al., 2025).

Traditional methods for identifying tomato fruits in greenhouse environments have mainly relied on the extraction and analysis of color and shape features. For example, Kurtulmuş et al. (2011) proposed an algorithm for detecting unripe citrus fruits using Eigenfruit, a technique that combines principal component analysis (PCA) with color and texture features, along with circular Gabor texture features to capture fine surface patterns. This method achieved a detection rate of 75.3% under natural outdoor conditions, but its performance degraded under extreme lighting variations or when the fruit’s color closely matched the background. Lin et al. (2020) developed an algorithm integrating color, depth, and shape information to detect spherical or cylindrical fruits, such as tomatoes and citrus, in natural environments. This method uses local shape matching for identifying contours and the probabilistic Hough transform to detect circular features. By combining these techniques, the algorithm improves robustness in detecting fruits that are partially occluded, especially in complex natural scenes. Wan et al. (2018) proposed a method for fresh tomato maturity detection using HSV color space for segmentation and edge detection for shape analysis. The method demonstrated promising performance in controlled settings; however, it faced challenges when applied to environments with complex backgrounds, occlusions, and fruits with irregular shapes. In conclusion, traditional image processing techniques rely on fixed features, which hinder their robustness when exposed to significant lighting variations or complex occlusions. Additionally, these methods often struggle with low computational efficiency, making them inadequate for meeting the dual demands of high accuracy and real-time performance required by contemporary agricultural robotics.

Deep convolutional neural networks (CNNs) (Deng et al., 2009) have currently become the mainstream technical paradigm for tomato target detection tasks in greenhouse scenarios. The existing tomato detection methods based on this network can mainly be classified into two-stage detection and single-stage detection two technical frameworks. Two-stage methods (region-based), exemplified by R-CNN (Girshick et al., 2014), Fast R-CNN (Girshick, 2015), and Faster R-CNN (Ren et al., 2015), operate by generating region proposals prior to classification. Although highly accurate, their computational complexity entails significant latency, often precluding real-time deployment. Conversely, regression-based single-stage models, notably the YOLO series (Redmon et al., 2016), execute simultaneous localization and classification, thereby offering a more efficient solution for rapid inference. Dong et al. (2025) proposed a lightweight GPC-YOLO model based on YOLOv8n, incorporating the PConv-based C2f-PC module and SimAM attention mechanism, which improved detection accuracy to 98.7%. Liang et al. (2025) focused on optimizing the network’s neck structure by using the Slim-Neck design and integrating the EMA attention mechanism to enhance feature representation. The resulting model achieved an mAP@50 of 96.4%, effectively improving the efficiency of automated cherry tomato harvesting. Wang and Liu (2025) introduced the TomatoGuard-YOLO framework, innovatively designing Multi-Path Inverse Residual Units (MPIRU) and a Dynamic Focused Attention Framework (DFAF), significantly enhancing multi-scale feature extraction performance. The model achieved 94.23% mAP@50 and 129.64 FPS, demonstrating a balance between accuracy and speed.Chai et al. (2025) explored multimodal fusion with the DCFA-YOLO network, which integrates RGB and depth information using a dual-channel cross-feature fusion mechanism. The backbone network was optimized with ShuffleNetV2 units, achieving 96.5% mAP for tomato cluster detection. However, the inclusion of depth information increased hardware costs and processing complexity, limiting its widespread deployment on low-cost edge devices. Additionally, Wang A. et al. (2025) proposed the PC-YOLO11s model, which strengthens the network’s ability to capture small object spatial and location information by adding the P2 layer, removing the P5 layer, and incorporating a coordinate space attention mechanism. Gao et al. (2025) focused on optimizing modules and loss functions, embedding gated convolutions in the C3K2 module and using the Wise-Powerful intersection over union (Wise-PIoU) loss function, achieving a model accuracy of 94.2%. In summary, while existing studies have made breakthroughs in improving accuracy or reducing computational overhead, no comprehensive solution has been developed that achieves high precision and robustness on edge devices while adapting to complex greenhouse environments.

In real-world greenhouse environments, rapid and accurate tomato fruit recognition is significantly challenged by factors such as fruit overlapping, severe occlusion, and variations in size and spatial distribution. To address these issues, a novel lightweight model, YOLO-FLBM, is proposed. The design objective of this model is to achieve efficient and accurate recognition in complex greenhouse environments while maintaining a lightweight structure. This approach aims to overcome the limitations present in current research and provide a solid technical foundation for the vision systems of harvesting robots. This study focuses on the object detection challenges encountered in automated tomato harvesting, with the primary goal being the development and optimization of the YOLO-FLBM model to enhance its detection accuracy in complex environments, while ensuring both efficiency and compactness. The key contributions of this study are summarized as follows:

1. The lightweight FasterNet architecture was specifically optimized and used to replace the original backbone network. The last three layers of the original FasterNet were removed, allowing the network to directly output feature maps that contain both rich semantic information and fine spatial details;
2. An innovative neck architecture, designated as the LB Neck, was devised by employing a dual-optimization strategy. On one hand, a novel C2f-LS module was designed to further compress the parameter size while preserving robust feature representation capabilities. On the other hand, the BiFPN structure was integrated to facilitate efficient bidirectional weighted feature fusion, thereby enhancing the information interaction among multi-scale features.
3. To address the challenges of occlusion and scale variation, a novel multi-scale coordinate dynamic attention (MCDA) mechanism was innovatively integrated into the network. By combining hybrid perception pooling with full-rank kernel generation, MCDA enables the model to dynamically capture spatial positions and long-range dependencies, effectively reducing missed detections caused by severe occlusion and scale variations;
4. The proposed YOLO-FLBM model demonstrates superior overall performance, achieving an optimal balance between accuracy and efficiency. The final model attains a precision rate of 95.2% and a mAP@50 of 97.4%, surpassing the baseline YOLOv8s model by 3.7% and 1.9%, respectively. With its compact parameter size and high detection accuracy, YOLO-FLBM is well-suited for deployment on agricultural edge devices for real-time tomato harvesting tasks.

## 2 Materials and data

### 2.1 Data acquisition

**Figure 1:**
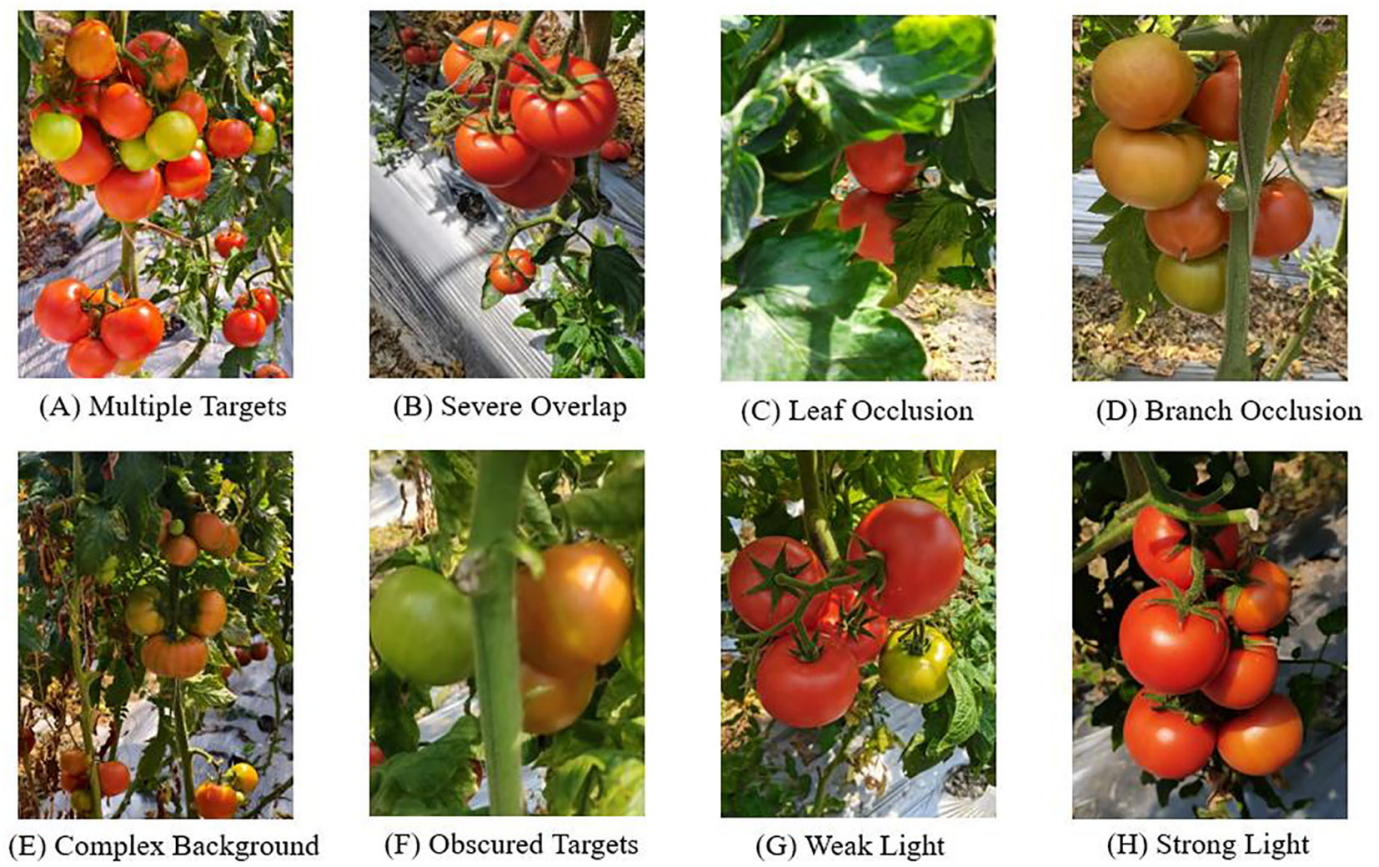
Images of tomatoes under different conditions. **(A)** Multiple Targets. **(B)** Severe Overlap. **(C)** Leaf Occlusion. **(D)** Branch Occlusion. **(E)** Complex Background. **(F)** Obscured Targets. **(G)** Weak Light. **(H)** Strong Light.

The tomato dataset was collected from Heshang Town, Changle District, Fuzhou City, Fujian Province, China (longitude 119.54883° E, latitude 25.93867° N). Image capture was conducted using iPhone and Huawei cameras, with the subjects positioned 0.3 to 0.6 meters from the camera. The images were captured on March 23, 2025, between 9:00 AM and 4:00 PM. A total of 788 images of tomatoes at various ripeness stages were collected in JPG format. The images were taken under natural light conditions and include multiple target images, overlapping fruit images, high-light images, low-light images, images with tomato leaf occlusion, images with tomato stem occlusion, blurred images, and images with complex backgrounds. **Figure 1** shows a selection of images taken under different conditions.

In this study, the LabelImg tool was used to manually annotate tomato images. The annotation data for each image were stored in an extensible markup language (TXT) file format, following the YOLO format. To meet the training requirements of the detection model, the images were resized to a uniform dimension of 640×640 pixels and converted to RGB three-channel images. Based on the color changes occurring during the ripening process, the tomatoes were categorized into three distinct ripening stages: ripe, indicating tomatoes with fully changed color; semi-ripe, indicating tomatoes that have started but not yet completed the color change; and unripe, corresponding to tomatoes that have not undergone any color change. The original dataset consisted of 788 images containing 6,973 annotated tomato instances. Following the data augmentation procedures, the total number of annotated targets was expanded to 27,812.

The dataset for tomato maturity detection constructed in this paper consists of 788 original images, all of which were captured in a real greenhouse environment. Although the number of original images is relatively limited, during the data collection process, typical complex factors in the detection of greenhouse tomatoes were as much as possible covered, including dense fruit distribution, leaf shading, fruit overlap, background interference, different maturity stages, and natural light changes. Therefore, this dataset can to some extent reflect the actual characteristics of the tomato maturity detection task in complex greenhouse environments.

### 2.2 Data augmentation

**Figure 2:**
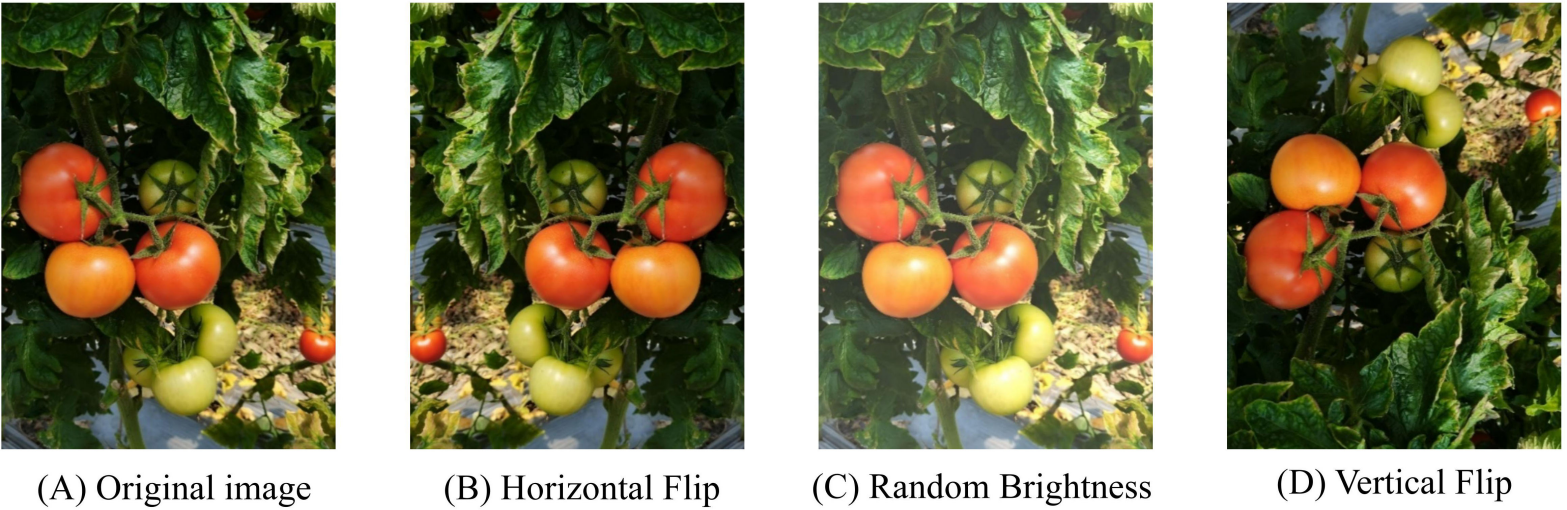
Data augmentation examples. **(A)** Original image. **(B)** Horizontal Flip. **(C)** Random Brightness. **(D)** Vertical Flip.

To enhance the model’s generalization ability in complex greenhouse environments and effectively mitigate overfitting, offline data augmentation was applied to the dataset using the Albumentations library (Buslaev et al., 2020). In selecting the data augmentation methods, this study focused on preserving key visual features related to tomato ripeness while avoiding the introduction of unnatural geometric distortions. Specifically, each original image underwent three operations: vertical flipping, horizontal flipping, and brightness adjustment, with the augmentation effects illustrated in **Figure 2**. The brightness adjustment was employed to simulate variations in light intensity caused by greenhouse shading, thereby improving the model’s adaptability to lighting conditions. Horizontal and vertical flipping were used to simulate imaging differences at various angles of detection and the diverse growth orientations of tomato fruits, enhancing the model’s robustness to changes in target orientation. As a result, the dataset size was expanded to 3,152 images, approximately four times the original data volume, providing a solid data foundation for the model’s comprehensive training. The dataset was divided into two subsets with an 8:2 split for training and validation.

## 3 Methods

### 3.1 YOLO-FLBM network structure

**Figure 3:**
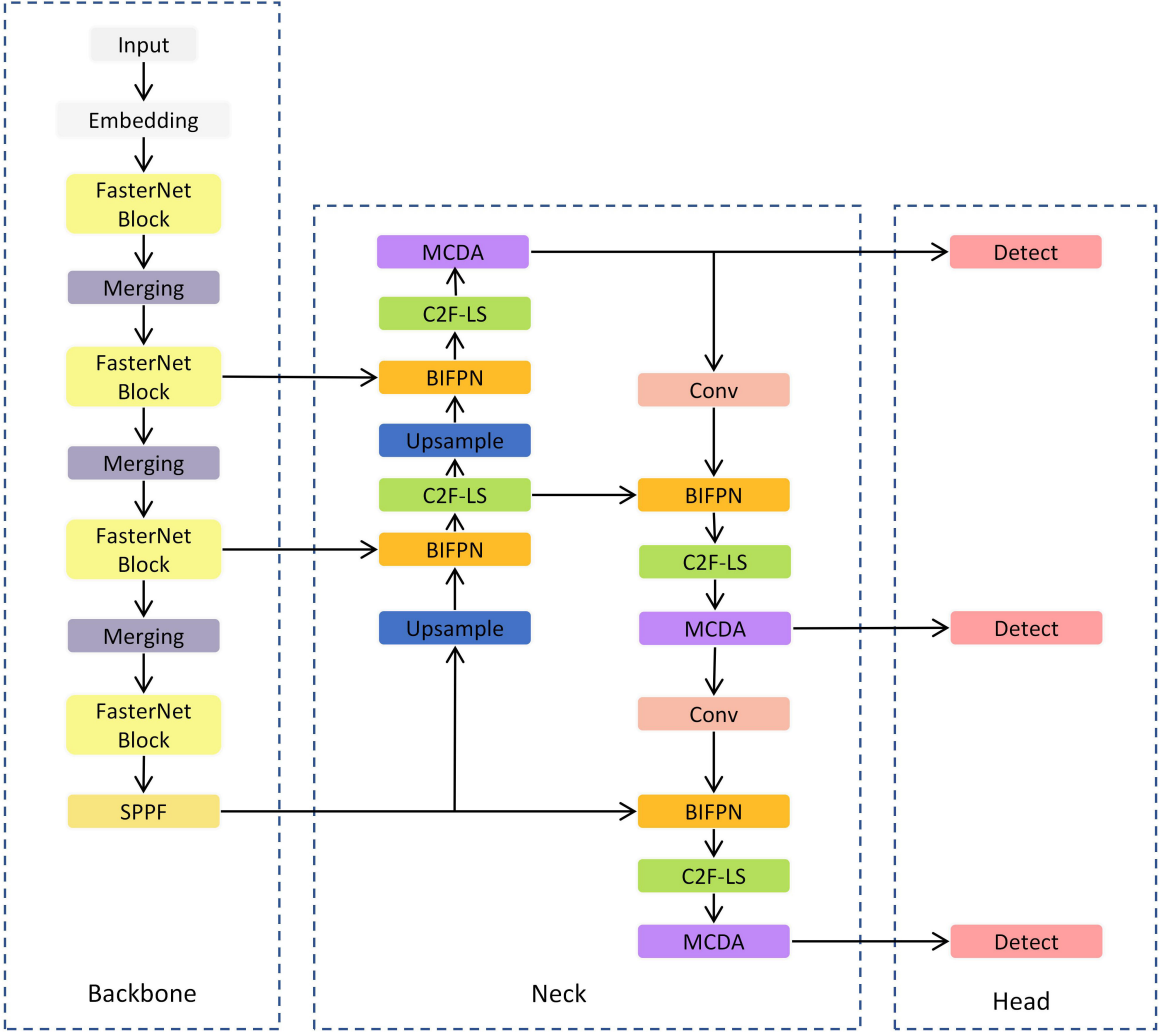
The architecture of the YOLO-FLBM model.

To address the conflict between limited computational resources and the need for high precision in greenhouse tomato detection tasks, a lightweight, high-performance detection model, YOLO-FLBM, was developed based on YOLOv8s. First, the backbone network was replaced with FasterNet to enhance model performance while effectively reducing the number of parameters. Next, a novel neck architecture, designated as the LB neck, was developed by integrating the BiFPN and C2f-LS modules. BiFPN facilitates bidirectional weighted fusion of multi-scale features, while the C2f-LS module increases the effective receptive field while compressing the parameters. Finally, the MCDA attention mechanism was embedded in the critical path of the neck, significantly improving the model’s ability to distinguish occluded and overlapping targets through full-rank dynamic kernel generation and a hybrid perception strategy. The improved network architecture is shown in **Figure 3**.

### 3.2 FasterNet

FasterNet (Chen et al., 2023) is a well-designed lightweight convolutional neural network aimed at maximizing inference speed and effectively reducing computational latency, without sacrificing feature extraction accuracy. The network incorporates the innovative Partial Convolution (PConv) technique, which exploits spatial redundancy in feature maps. This approach significantly reduces the number of floating-point operations while effectively addressing the latency issues caused by frequent memory accesses in traditional depthwise separable convolutions (DWConv).

**Figure 4:**
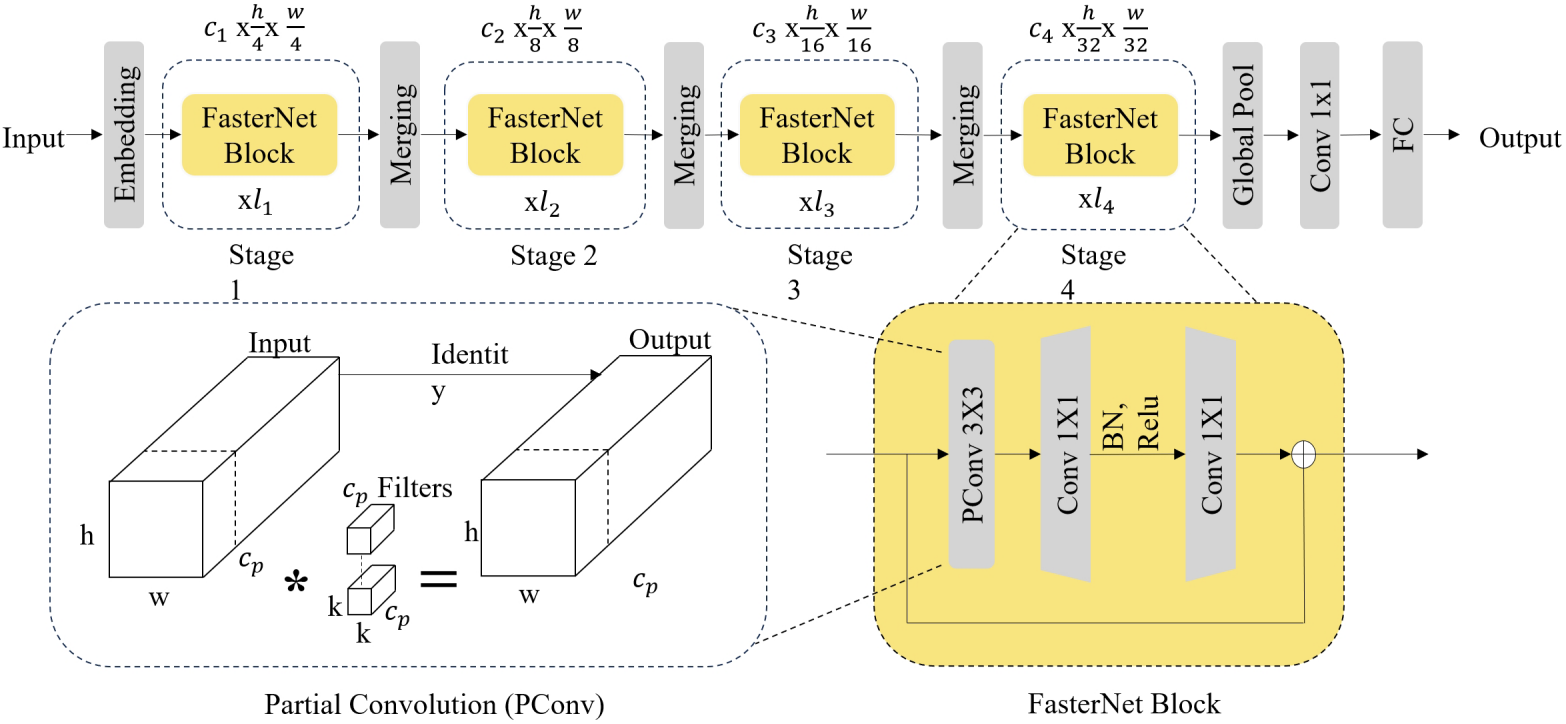
Overall architecture of FasterNet

The overall architecture of FasterNet consists of four hierarchical stages, with each stage comprising a series of stacked FasterNet Blocks and preceding embedding or merging layers, designed for hierarchical feature extraction. Each FasterNet Block contains one PConv layer and two PWConv layers, forming an inverted residual structure. This structure effectively leverages the information from all channels, creating a T-shaped convolution structure that allows the model to focus more on the features in the central region. After the PConv operation, normalization and activation layers are added only after the middle layer, preserving feature diversity and reducing computational latency. The architecture of FasterNet is shown in **Figure 4**.

To efficiently integrate FasterNet into the YOLO detection framework, targeted adjustments were made to its original architecture. The global average pooling, 1×1 convolution, and fully connected layers at the end of FasterNet were removed. These specific components (Global Average Pooling, 1×1 Conv, and FC layer) constitute the original classification head of FasterNet. We removed them to ensure that the backbone outputs high-resolution feature maps suitable for spatial localization in tomato detection, rather than a single classification vector. This not only optimized the multi-scale feature fusion efficiency of the subsequent neck network but also significantly reduced computational redundancy and parameter size. This improvement strategy, while achieving model compression, effectively prevented excessive feature compression and greatly enhanced the precise localization of tomato fruits in complex backgrounds.

### 3.3 LB neck architecture

To simultaneously address the challenges of complex occlusion, significant scale variations of tomato fruits, and the strict requirements for model compression in greenhouse environments, a novel neck architecture, designated as the LB neck, was constructed. This architecture architecturally integrates the proposed C2f-LS module with the BiFPN structure to achieve efficient parameter compression while strengthening multi-scale feature fusion capabilities.

**Figure 5:**
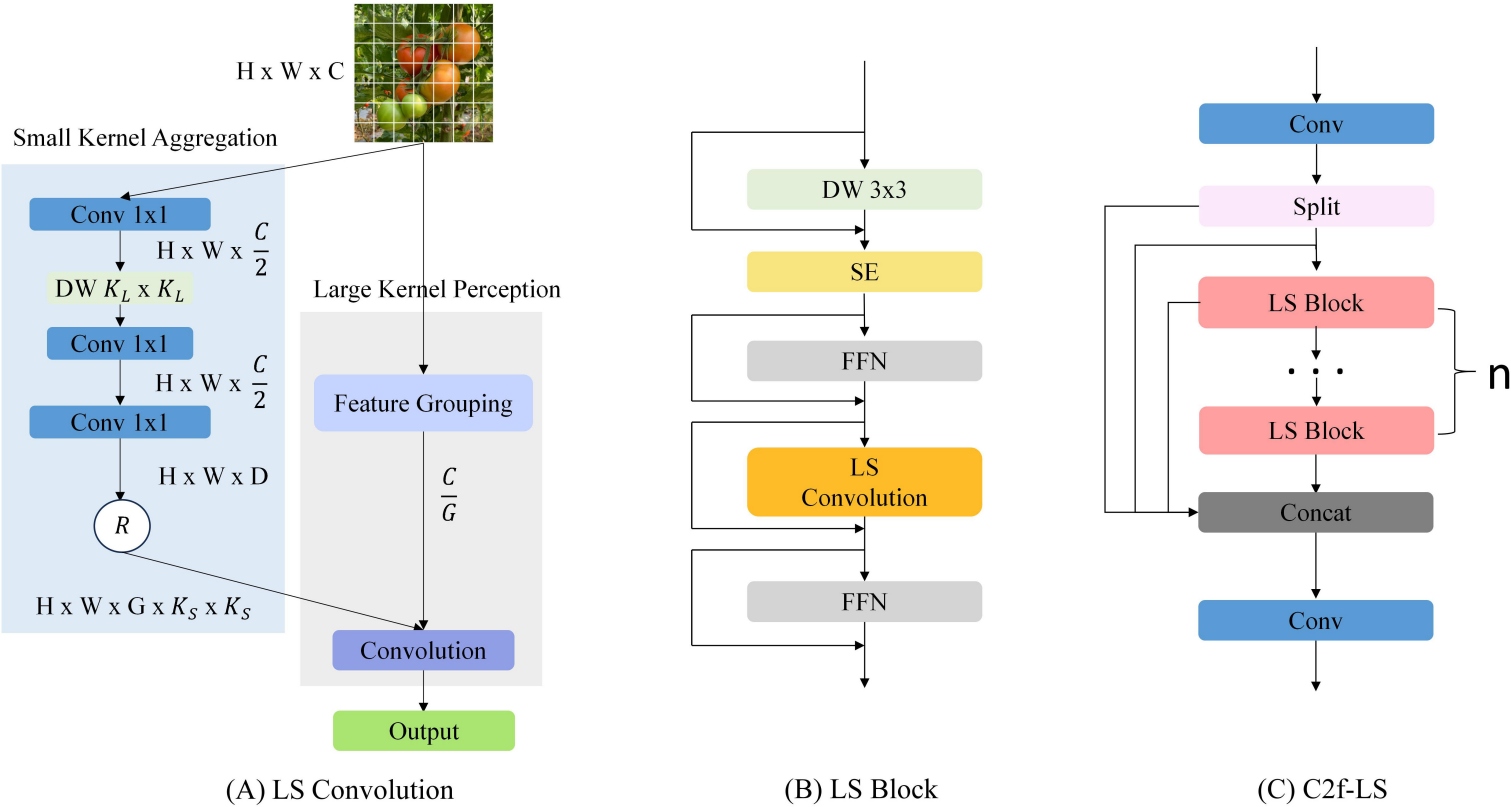
**(A)** The illustration of LS convolution. **(B)** The illustration of LS Block. **(C)** The illustration of C2f-LS.

The C2f-LS module was developed to overcome the limitations of the standard C2f module, where the receptive field is constrained by 3x3 convolutions, hindering the capture of long-range dependencies in obstructed tomato clusters. While retaining the efficient gradient flow advantages of the Cross-Stage Partial (CSP) network, the C2f-LS module replaces the internal bottleneck layer with a lightweight LS Block (Large Selective Block) (Wang Z. et al., 2025). Inspired by the “wide-area perception and local focus” mechanism of human vision, the LS Block (Large Selective Block) [22] is designed to balance computational efficiency with an effective receptive field. As illustrated in **Figure 5**, the module adopts a sequential structure: the initial stage captures local textures and channel dependencies utilizing a 3x3 depthwise separable convolution combined with the SE channel attention mechanism. Subsequently, the core LS Convolution is introduced, comprising two complementary branches: Large Kernel Perception (LKP) and Small Kernel Aggregation (SKA). The LKP branch utilizes downsampled large-kernel depthwise convolutions (e.g., 7x7) to capture broad spatial context and generate adaptive weights, which are then applied by the SKA branch to perform dynamic convolutions on grouped features using small kernels (e.g., 3x3). This design effectively simulates the wide field of view characteristic of large convolutional kernels without significantly increasing the computational burden.

**Figure 6:**
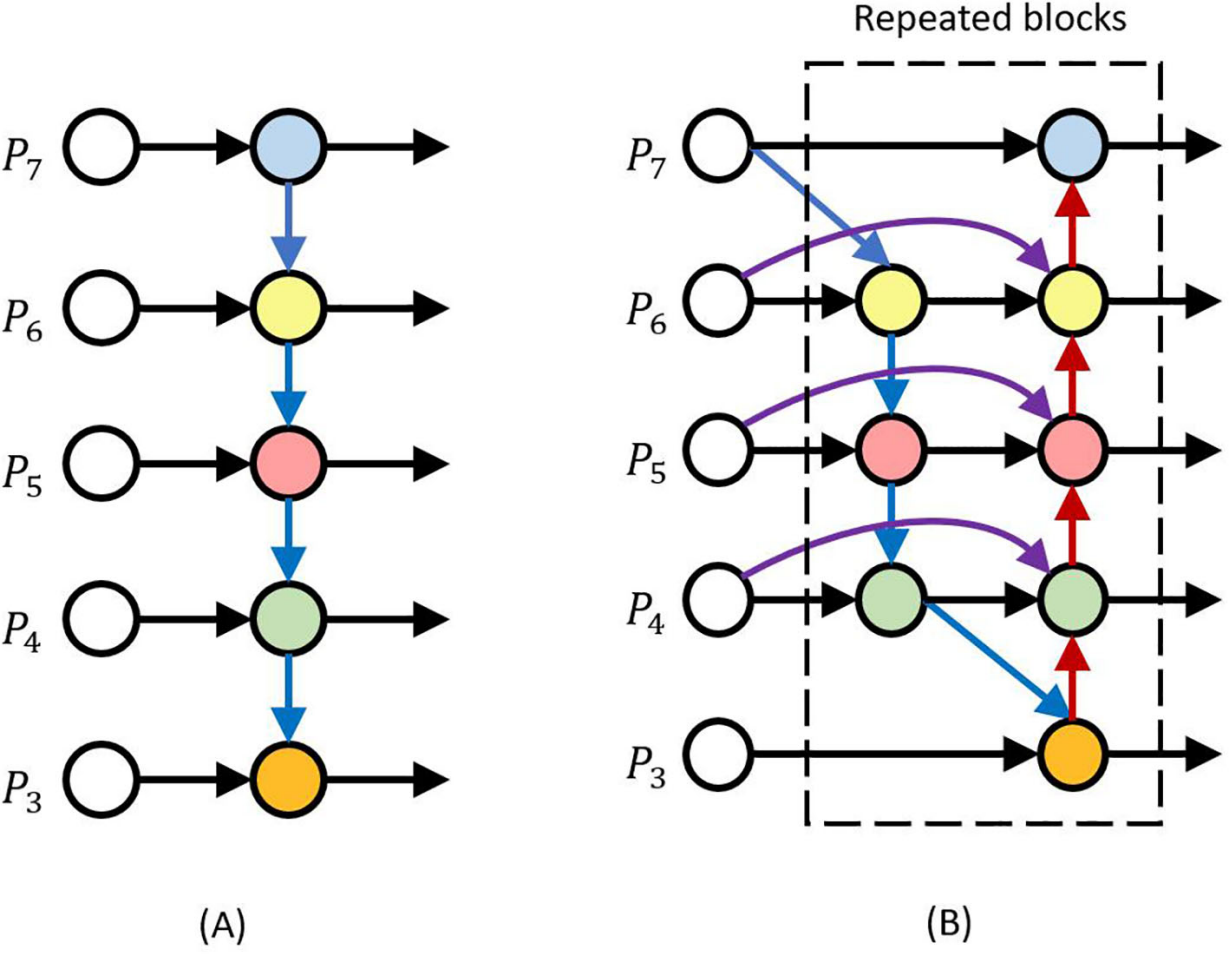
**(A)** FPN introduced a top-down pathway for fusing multi-scale features from levels 3 to 7 (P3-P7); **(B)** BiFPN, which achieves a better balance between accuracy and efficiency.

Complementing the enhanced feature extraction of C2f-LS, the BiFPN (Bidirectional Feature Pyramid Network) (Tan et al., 2020) structure was incorporated to restructure the neck network. Given the significant scale variations of tomato fruits—ranging from immature small fruits to fully mature large fruits—the traditional PANet (Liu et al., 2018) architecture is limited by its strategy of assigning equal weights to feature maps of different resolutions. To address this, BiFPN introduces bidirectional skip connections across scales and a learnable weight mechanism, as shown in **Figure 6**. This allows the network to adaptively adjust the fusion ratios of the P3, P4, and P5 feature layers during training, ensuring that shallow features rich in texture and deep features rich in semantics are optimally combined. By synergizing the receptive field expansion of C2f-LS with the adaptive fusion of BiFPN, the LB neck significantly enhances the model’s robustness and detection accuracy for small and occluded tomato targets.

### 3.4 Multi-scale coordinate dynamic attention

In the field of deep learning, attention mechanisms, by simulating the human visual focusing process, have significantly enhanced the model’s ability to represent complex environments. However, in the task of greenhouse tomato detection, challenges arise due to severe occlusion by branches and leaves, dense overlapping of fruits, and scale variations resulting from different growth stages, all of which complicate feature extraction. While traditional dynamic convolutional attention mechanisms possess content-awareness, they are often limited by the use of global average pooling to generate weights, which leads to the loss of critical spatial location information. Furthermore, their single convolutional receptive field struggles to capture both the fruit’s surface texture and long-range contour context, thus restricting the model’s discriminative accuracy in complex backgrounds. To address these recognition challenges, we propose the Multi-scale Coordinate Dynamic Attention (MCDA) mechanism.

**Figure 7:**
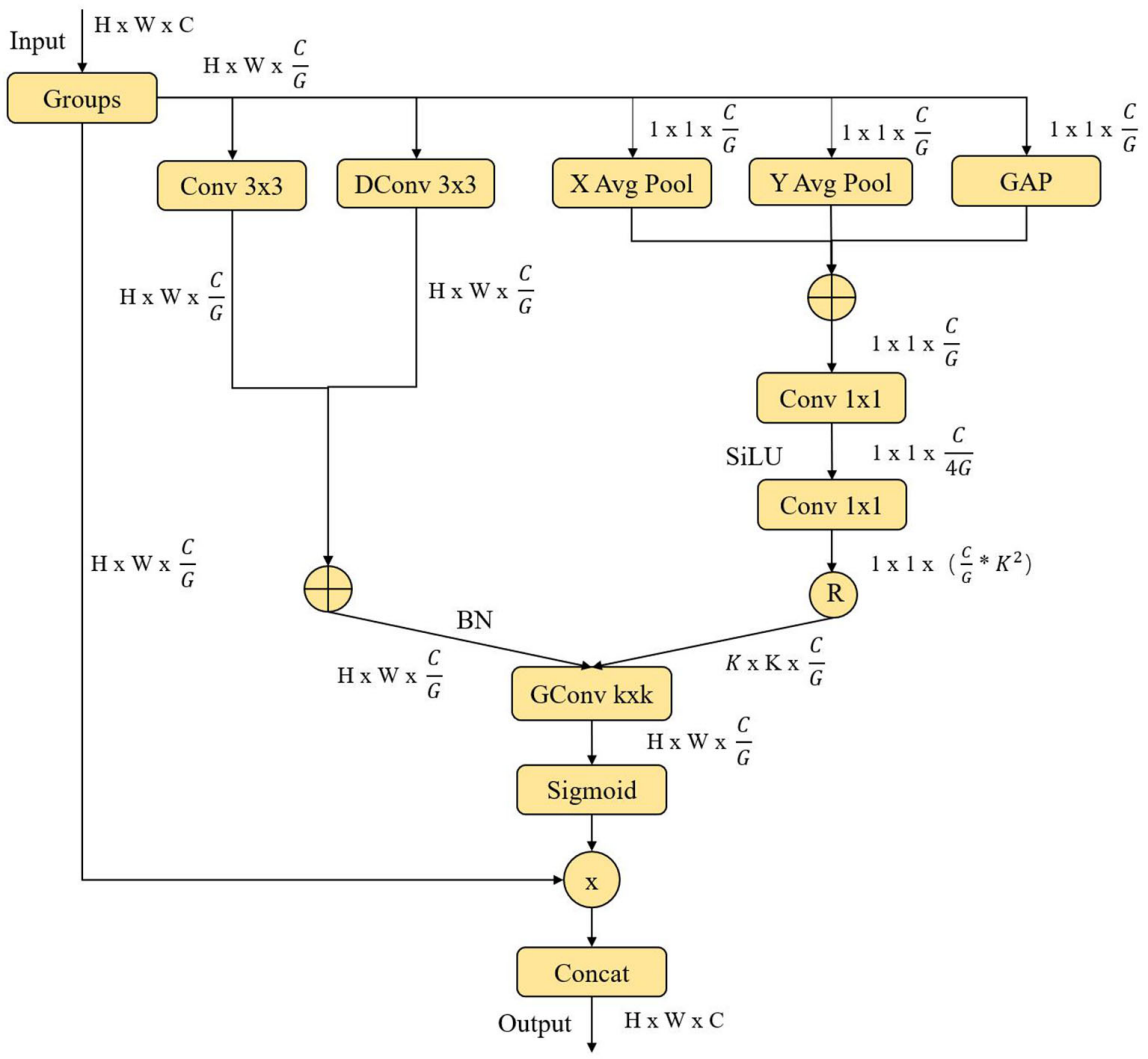
MCDA module structure.

MCDA innovatively constructs a parallel dual-stream architecture, incorporating hybrid perception kernel generation and multi-scale feature aggregation. The module first reduces computational complexity using a channel grouping strategy, and then independently performs feature interactions within each subgroup. By endowing the dynamic kernel with “positional awareness” and “multi-scale vision, “ adaptive recalibration of complex features is achieved. The architecture of the MCDA module is illustrated in **Figure 7**.

In the kernel generation branch, MCDA introduces a hybrid perception pooling strategy to construct context descriptors with positional sensitivity. Unlike traditional methods that rely solely on global average pooling (GAP), this branch performs parallel coordinate pooling operations along the horizontal (X-axis) and vertical (Y-axis) directions, with the outputs of the three operations fused element-wise. For a given input feature map $\text{X}\in {\text{R}}^{\text{C}\times \text{H}\times \text{W}}$, the hybrid context descriptor ${\text{Z}}_{\text{C}}\in {\text{R}}^{\text{C}\times 1\times 1}\;$is formulated as:


$${\text{Z}}_{\text{C}}={\text{Pool}}_{\text{gap}}(\text{X})+\text{Mean}({\text{Pool}}_{\text{h}}(\text{X}))+\text{Mean}({\text{Pool}}_{\text{ω}}(\text{X}))$$

Where ${\text{Pool}}_{\text{gap}}$denotes the standard global average pooling, and ${\text{Pool}}_{\text{h}}$ and ${\text{Pool}}_{\text{ω}}$ perform 1D adaptive average pooling along the spatial axes.This design not only preserves the semantic stability of global categories but also explicitly encodes long-range spatial dependencies, enabling the generated dynamic kernel to perceive the precise location of the target. The fused context features are then passed through a lightweight multilayer perceptron (MLP) consisting of two 1×1 convolutional layers. The features are first downsampled to reduce parameter redundancy and then upsampled to regenerate the full-rank dynamic convolution kernel parameters. Formally, the full-rank dynamic kernel θ is generated by:


$$\text{θ}={\text{Conv}}_{\text{exp}}(\text{SiLU}({\text{Conv}}_{\text{red}}({\text{Z}}_{\text{c}})))$$

Where ${\text{Conv}}_{\text{red}}$ ${\text{Conv}}_{\text{exp}}$ represent the channel reduction and expansion convolutions, respectively. This full-rank generation mechanism prevents feature expression loss caused by low-rank decomposition, ensuring that the generated filters can accurately model complex textures and irregular boundaries.

In the feature extraction branch, MCDA employs an additive multi-scale aggregation strategy to enhance the richness of tomato features. The input features are processed through two parallel convolutional paths: one path uses standard 3×3 convolutions to focus on extracting high-frequency local details, such as the fruit’s surface texture; the other path introduces dilated convolutions with a dilation rate of 2, expanding the receptive field without increasing the number of parameters, in order to capture the contextual relationship between the fruit and its surrounding branches and leaves. The outputs of the two paths are fused through addition rather than simple concatenation. This additive multi-scale feature extraction can be mathematically expressed as:


$$F_{base}=BN(DWConv_{3\times 3}(X)+Dilated-DWConv_{3\times 3,d=2}(X))$$

Where  DWConv denotes depthwise convolution and BN represents batch normalization. This ensures that each feature channel integrates both “local fine” and “global broad” visual information, significantly improving the model’s ability to represent targets of varying scales and those that are occluded.

Finally, the module achieves interaction between the two branches through dynamic grouped convolutions (GConv): the dynamic kernels, enriched with positional and semantic information, are used to filter the fused multi-scale feature maps. This process dynamically adjusts the filter weights based on the specific content of the input image, effectively enhancing the response values in the target regions while suppressing background noise. The convolution output is passed through a Sigmoid activation function to generate the attention weight map, which is then element-wise multiplied with the original input features to complete the feature recalibration. This dynamic calibration process is defined as:


$$A=\sigma (F_{base}⊛\Theta )$$

Y=X⊗A

where ⊛ denotes the dynamic group convolution using the generated kernel θ, σ is the Sigmoid activation function, ⊗ represents element-wise multiplication, and Y is the recalibrated output. The processed subgroups are ultimately concatenated along the channel dimension, resulting in a high-discriminative enhanced feature map that provides robust feature support for the subsequent detection head. Through this mechanism, MCDA generates high-discriminative feature maps, delivering precise feature support for multi-scale, occluded, and overlapping tomato targets.

## 4 Results and discussion

### 4.1 Experimental environment

To ensure the reproducibility and reliability of the experimental results, the experimental environment and key parameter settings were carefully documented in this study.

The experiments were conducted on a high-performance computing platform to ensure the efficiency of model training and inference. The hardware configuration includes an Intel^®^ Xeon^®^ Silver 4214R CPU (with a clock speed of 2.40 GHz) and the powerful NVIDIA GeForce RTX 3080Ti GPU (12 GB), which offers substantial parallel computing capabilities. The deep learning framework used was PyTorch 2.1.2, running in a Python 3.10 environment. To fully leverage GPU acceleration and optimize neural network performance, CUDA 11.8 was configured, providing strong support for the training process.

**Figure 8:**
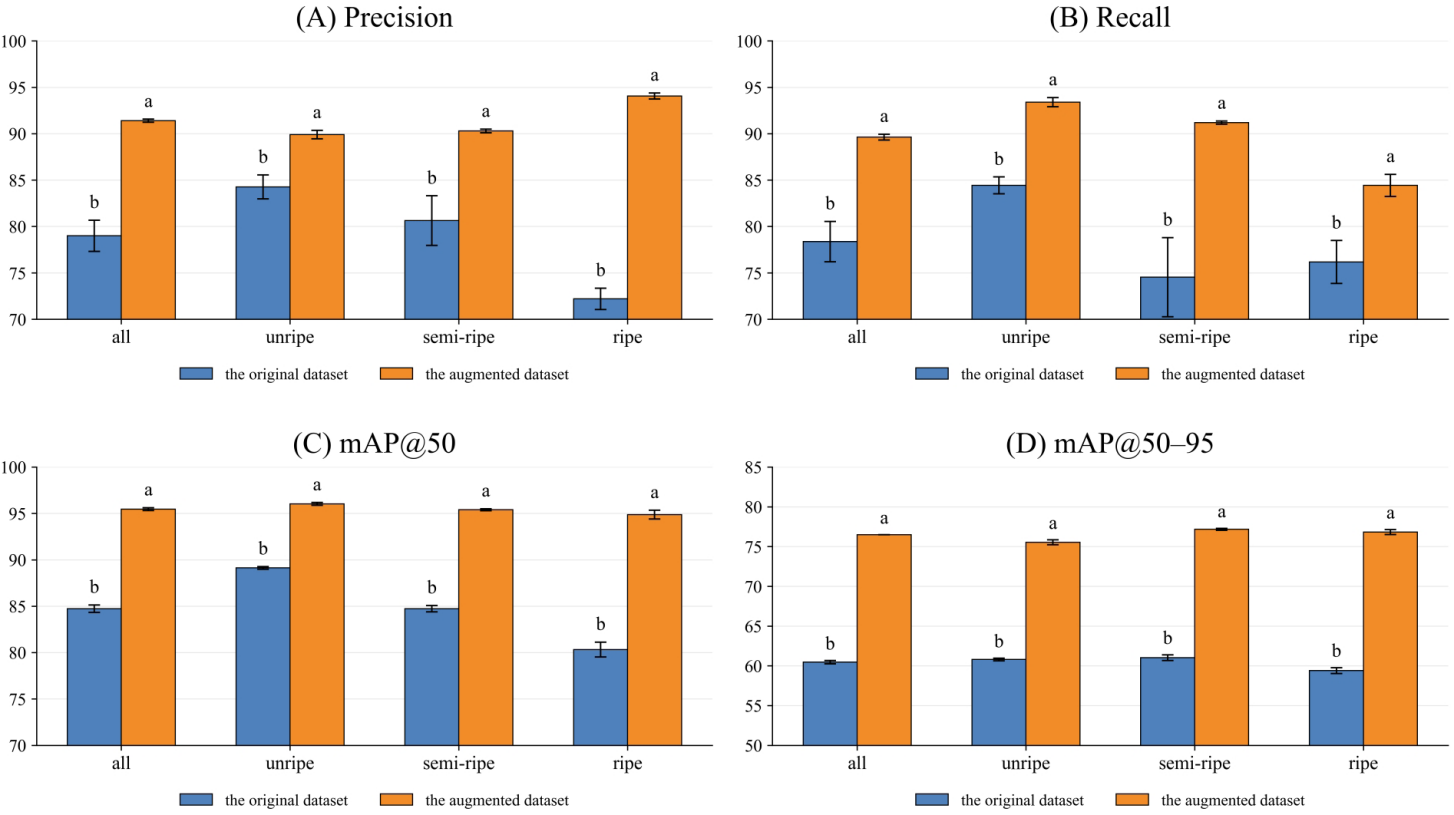
Performance comparison of YOLOv8s trained on the original and augmented datasets for detecting tomatoes at different ripening stages. **(A)** Precision. **(B)** Recall. **(C)** mAP@50. **(D)** mAP@50-95. Data are presented as mean ± standard deviation from three independent runs with different random seeds. Different lowercase letters above the bars indicate significant differences between the original and augmented datasets at p < 0.05.

**Figure 9:**
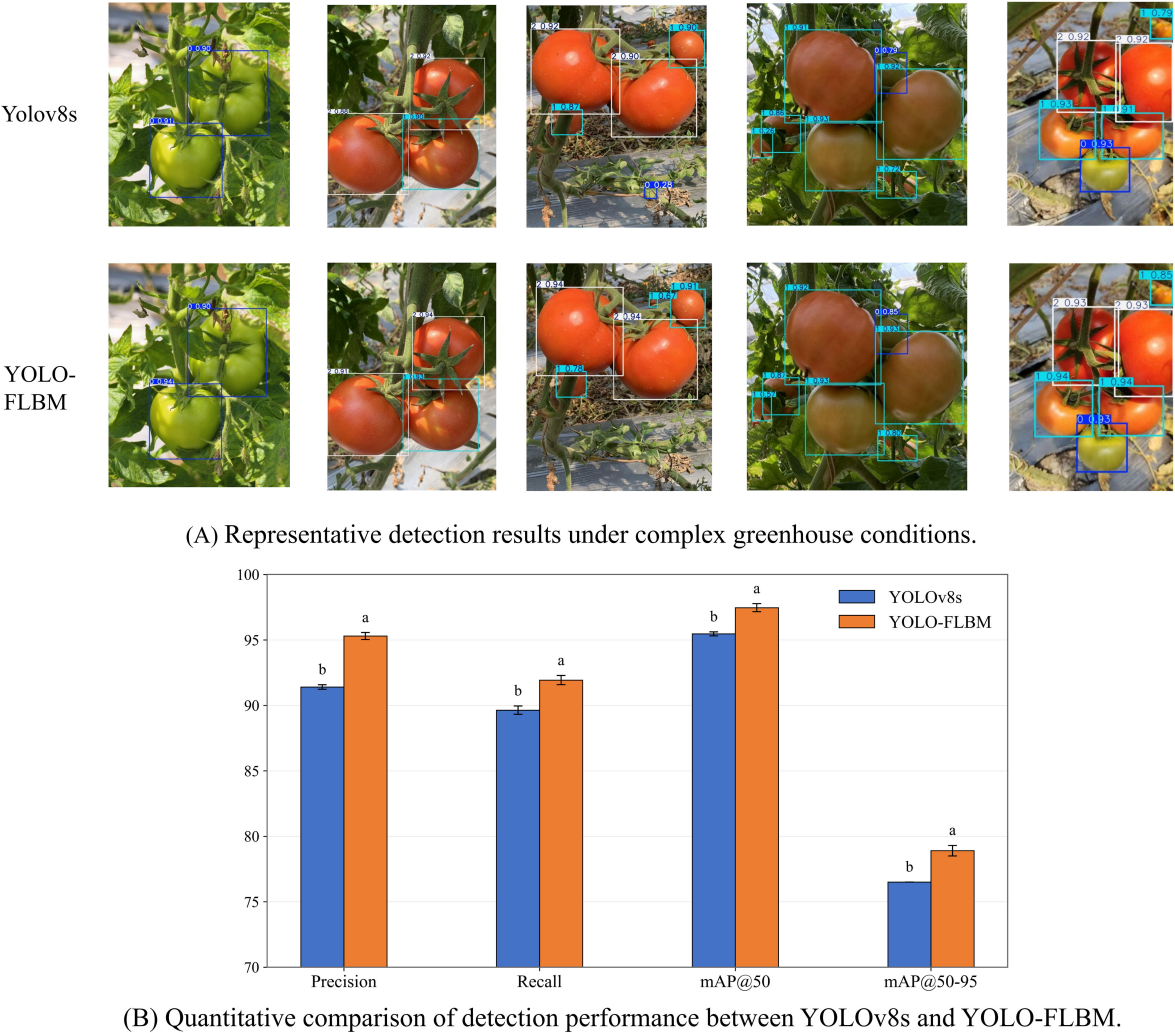
Qualitative and statistical comparison between YOLOv8s and YOLO-FLBM. **(A)** Representative detection results under complex greenhouse conditions. **(B)** Quantitative comparison of detection performance between YOLOv8s and YOLO-FLBM. Data are presented as mean ± standard deviation from three independent runs. Different lowercase letters indicate significant differences between the two models at p < 0.05.

Regarding the training strategy, specific hyperparameters were chosen to balance the model’s convergence speed and generalization ability. The training process consisted of 200 epochs, with a batch size of 16, selected to accommodate the GPU memory limitations while ensuring stable gradient estimation. The Adam optimizer was used for parameter updates, with an initial learning rate set to 0.01. Additionally, a weight decay of 0.0005 was applied to mitigate potential overfitting during the training phase. Unless otherwise specified, the results reported in the comparative tables correspond to representative runs under the default experimental setting. For the statistical analyses in **Figures 8** and **9**, experiments were independently repeated three times using different random seeds, and the error bars represent the mean ± standard deviation.

Although the model was trained on a high-performance workstation, practical agricultural applications require deployment on resource-constrained edge devices. Thus, the Rockchip RK3588 embedded platform was utilized to evaluate edge inference performance, with its hardware configurations detailed in **Table 1**. The lightweight design of the proposed model is directly driven by the specific technical limitations of this hardware. While the onboard Neural Processing Unit (NPU) offers a peak throughput of 6 TOPS, this computational capacity must be shared with concurrent system operations in actual robotic frameworks. Additionally, the device operates within a limited 8GB RAM environment. This restricted memory footprint is dynamically allocated among high-resolution image buffers, model activation maps, and background operating system tasks. These hardware bottlenecks—specifically the bounded NPU throughput and memory capacity—strictly limit the permissible model complexity. Therefore, minimizing the parameter count and computational overhead (FLOPs) is essential to achieve stable, real-time edge inference without causing resource depletion.

**Table 1:** Hardware specifications of the embedded deployment platform.

Hardware environment	Configurations
Embedded Board	Rockchip RK3588 Development Board
CPU	Octa-core processor combining 4 × Cortex-A76 and 4 × Cortex-A55 cores, with a peak clock speed of 2.4 GHz.
NPU	Embedded AI accelerator (NPU) with a 6 TOPS peak throughput
Camera	MCIMX415 industrial-grade imaging sensor with high-definition resolution
Screen	5.5-inch MIPI-interface terminal supporting 1920×1080 definition

### 4.2 Evaluating indicators

In this study, mean Average Precision (mAP), precision, recall, F1 score, floating-point operations per second (GFLOPs), model parameters, and model size (MB) were selected as performance metrics for evaluating the deep learning model. These evaluation metrics are computed using the following formulas:


$$\text{Precision}=\frac{\text{TP}}{\left(TP+FP\right)}$$

$$\text{Recall}=\frac{\text{TP}}{\left(\text{TP}+\text{FN}\right)}$$

$$\text{m}AP=\frac{\;\sum_{i=1}^{n}{\text{AP}}_{i}}{\text{n}}$$

$$\text{F}1=\frac{2\times Precision\times \text{Recall}}{Precision+Recall}$$

In this study, TP represents the number of images in which the model correctly detected tomato fruit targets, FP represents the number of images in which the model incorrectly detected non-tomato fruit targets, and FN represents the number of images in which the model failed to detect tomato fruit targets. The F1 score is the harmonic mean of precision and recall, providing a balanced evaluation of these metrics, which is particularly useful when precision and recall are imbalanced. Precision reflects the accuracy of the model’s positive predictions, while recall measures the model’s ability to cover true positive samples. The values of precision and recall are used to construct the precision-recall curve (PR curve), with the area under the curve representing the average precision (AP). The mean Average Precision (mAP) represents the average of AP values and is used to evaluate the overall performance of the model in multi-class detection tasks. The model parameters are calculated based on the number of input and output channels, as well as the convolution kernel size, providing an estimate of the model size. GFLOPs are used to measure the computational complexity of the model. The model size (MB) corresponds to the disk space occupied by the weight files and impacts both the loading speed and memory consumption during execution.

By comprehensively evaluating these metrics, YOLO-FLBM demonstrates a well-balanced performance in terms of detection accuracy, computational efficiency, storage overhead, and practical deployment capability.

### 4.3 Performance of the data augmentation method

To assess the effect of data augmentation, YOLOv8s was trained and evaluated on both the original and augmented datasets under the same experimental settings. Each experiment was repeated three times with different random seeds, and the results are reported as mean ± standard deviation in **Figure 8**. For the original dataset, YOLOv8s achieved 79.00 ± 1.68% precision, 78.37 ± 2.17% recall, 84.73 ± 0.40% mAP@50, and 60.47 ± 0.21% mAP@50–95. At the class level, the precision values for unripe, semi-ripe, and ripe tomatoes were 84.27 ± 1.29%, 80.63 ± 2.68%, and 72.20 ± 1.15%, respectively. The corresponding recall values were 84.43 ± 0.91%, 74.53 ± 4.27%, and 76.17 ± 2.31%, respectively. For mAP@50, the three categories achieved 89.13 ± 0.15%, 84.73 ± 0.35%, and 80.33 ± 0.80%, while the mAP@50–95 values were 60.80 ± 0.17%, 61.03 ± 0.35%, and 59.40 ± 0.36%, respectively. After data augmentation, the overall precision, recall, mAP@50, and mAP@50–95 increased to 91.40 ± 0.17%, 89.63 ± 0.32%, 95.47 ± 0.15%, and 76.50 ± 0.00%, respectively. For unripe, semi-ripe, and ripe tomatoes, the precision values were 89.90 ± 0.46%, 90.30 ± 0.20%, and 94.07 ± 0.32%; the recall values were 93.40 ± 0.50%, 91.20 ± 0.17%, and 84.43 ± 1.19%; the mAP@50 values were 96.03 ± 0.15%, 95.40 ± 0.10%, and 94.87 ± 0.47%; and the mAP@50–95 values were 75.53 ± 0.31%, 77.17 ± 0.12%, and 76.83 ± 0.31%, respectively.

As shown in **Figure 8**, the model trained on the augmented dataset achieved higher mean performance than that trained on the original dataset across all evaluated metrics and ripeness categories. Statistical differences between the two datasets were analyzed using Student’s t-test, with the significance level set at p < 0.05. Different lowercase letters above the bars indicate significant differences. In addition to the improvement in mean performance, the augmented dataset generally resulted in smaller standard deviations, suggesting that data augmentation reduced the sensitivity of YOLOv8s to random seed variations. These results indicate that the adopted data augmentation strategy improved both the detection performance and training stability of YOLOv8s for tomato ripeness recognition.

### 4.4 Ablation experiments

Ablation experiments were conducted on the YOLOv8s model using our custom-built tomato dataset to evaluate the performance of the components integrated into the model, including FasterNet, C2f-LS, BiFPN, and MCDA. Starting with YOLOv8s, successive models progressively integrated the proposed improvements. Model 1 optimized the backbone network by incorporating the lightweight FasterNet architecture. Model 2 introduced the proposed C2f-LS module on top of Model 1. Model 3 replaced the original FPN structure with the BiFPN bidirectional feature pyramid network in Model 2. Finally, the proposed MCDA attention module was embedded into the neck network of Model 3, resulting in the YOLO-FLBM model. The experimental results are detailed in **Table 2**.

**Table 2:** Results of ablation experiment.

Model	FasterNet	LB neck:C2f-LS	LB neck : BiFPN	MCDA	P/%	R/%	mAP@50/%	mAP@50–95/%	Params/M
Yolov8s					91.5	89.4	95.5	76.5	9.828
Model1	✓				93.6	91.6	96.7	78.2	4.784
Model2	✓	✓			94.8	90.0	96.7	77.1	**3.501**
Model3	✓	✓	✓		94.8	91.3	97.2	78.0	3.501
YOLO-FLBM	✓	✓	✓	✓	**95.2**	**91.9**	**97.4**	**78.9**	3.743

* Bold values represent the optimal results for each metric.

As shown in **Table 2**, based on YOLOv8s, Model 1 achieved improvements across multiple metrics by integrating the lightweight FasterNet architecture. The precision, recall, mAP@50, and mAP@50–95 were increased by 2.1%, 2.2%, 1.2%, and 1.7%, respectively, while the model’s parameter count was reduced by 5.044M. The introduction of the C2f-LS module further enhanced the model’s precision by 1.2%, while also reducing the parameter count. However, this improvement resulted in a 1.6% decrease in recall and a 1.1% decrease in mAP@50-95. After the integration of BiFPN into Model 2, recall, mAP@50, and mAP@50–95 improved by 1.3%, 0.5%, and 0.9%, respectively, while maintaining the same precision and parameter count. This indicates that BiFPN not only preserved the lightweight advantages but also effectively addressed the observed decline in recall and mAP@50–95 in Model 2. With the addition of the MCDA attention module, compared to Model 3, precision, recall, mAP@50, and mAP@50–95 increased by 0.4%, 0.6%, 0.2%, and 0.9%, respectively, although the model’s parameter count slightly increased by 0.242M. This demonstrates the effectiveness of the MCDA attention mechanism in extracting tomato detection-related features.

In conclusion, the lightweight YOLO-FLBM model significantly outperforms the original YOLOv8s model. It not only achieves model compression but also maximizes improvements in detection accuracy. The model shows overall improvement in all metrics, with precision, recall, and mAP@50 increasing by 3.7%, 2.5%, and 1.9%, respectively. Moreover, the model’s parameter count was reduced by 61.91%, reaching 3.743M, demonstrating its efficiency and effectiveness in practical applications.

### 4.5 Lightweight network performance comparison

To further reduce model complexity while maintaining detection accuracy and meeting the real-time deployment requirements of edge devices, a comparative experiment was conducted under the same experimental conditions. The original backbone network of YOLOv8s was replaced with five mainstream lightweight networks: EfficientViT (Liu et al., 2023), MobileNetV3 (Howard et al., 2019), ShuffleNetV2 (Ma et al., 2018), GhostNet (Han et al., 2020) and FasterNet. The specific performance metrics are presented in **Table 3**.

**Table 3:** Lightweight network performance comparison.

Network	P/%	R/%	mAP@50/%	mAP@50–95/%	GFLOPs	Params/M
Yolov8s	91.5	89.4	95.5	76.5	23.5	9.828
EfficientViT	93.5	90.8	96.1	77.9	15.3	7.084
MobileNetV3	89.0	88.0	94.3	73.1	14.3	6.708
ShuffleNetV2	91.2	89.1	95.2	74.2	11.3	5.085
GhostNet	91.4	88.9	95.6	75.6	15.5	6.973
FasterNet	**93.6**	**91.6**	**96.7**	**78.2**	**11.1**	**4.784**

* Bold values represent the optimal results for each metric.

As shown in **Table 3**, the original YOLOv8s model achieved a mAP@50 of 95.5%, but its parameter count reached 9.828M, with a computational load of 23.5 GFLOPs. This high computational burden limits its inference speed on low-power platforms. Among the comparison models, EfficientViT showed improvements in accuracy and mAP@50, but its computational load and parameter count were 15.3 GFLOPs and 7.084M, respectively, offering no clear advantage over other lightweight networks. MobileNetV3 reduced computational costs but exhibited weak feature extraction capabilities, resulting in a decline in mAP@50 to 94.3%, which is insufficient for high-performance detection. ShuffleNetV2 demonstrated a more aggressive approach to lightweight design, with a parameter count of only 5.085M; however, its mAP@50 and mAP@50–95 slightly decreased, indicating a trade-off in feature extraction performance to achieve significant reductions in computational load. GhostNet achieved a slight increase in mAP@50 to 95.6% and reduced computational load by approximately 34% compared to the baseline model. However, its mAP@50–95 dropped to 75.6%, suggesting a compromise in overall detection performance.

In contrast, FasterNet exhibited the best overall performance. In terms of model compression, FasterNet’s parameter count was only 4.784M, representing a 51.3% reduction compared to the original YOLOv8s model, making it the smallest model among all compared networks. Its computational load was only 11.1 GFLOPs, significantly outperforming all other networks except ShuffleNetV2. More importantly, FasterNet maintained high detection performance while significantly reducing model size, achieving a comprehensive accuracy improvement. Its precision (P), recall (R), and mAP@50 reached 93.6%, 91.6%, and 96.7%, respectively, with the highest mAP@50–95 of 78.2% under high IoU thresholds. These results demonstrate that FasterNet, through more efficient convolution operations, effectively addresses the common issue of insufficient feature extraction capacity in lightweight networks, achieving the optimal balance between model complexity and detection accuracy.

### 4.6 Attention module comparative experiment

To further enhance the model’s ability to extract discriminative features of tomato fruits in complex backgrounds, various attention mechanisms were inserted at the same location in this study, including SE (Hu et al., 2018), ELA (Xu et al., 2025), EMA (Ouyang et al., 2023), CBAM (Woo et al., 2018), SimAM (Yang et al., 2021), and MCDA. The experimental results are shown in **Table 4**.

**Table 4:** Attention module comparative experiment.

Method	P/%	R/%	F1	mAP@50/%	mAP@50-95/%	Params/M
Model 3	94.8	91.3	0.930	97.2	78.0	3.501
Model 3+SE	93.2	**92.9**	0.926	96.9	77.3	3.544
Model 3+ELA	93.5	91.6	0.925	96.9	77.2	3.509
Model 3+EMA	94.4	91.7	0.930	97.1	77.5	3.556
Model 3+CBAM	92.7	91.0	0.918	96.3	76.2	3.846
Model 3+SimAM	94.4	91.1	0.927	97.1	77.8	**3.501**
Model 3+MCDA	**95.2**	91.9	**0.935**	**97.4**	**78.9**	3.743

* Bold values represent the optimal results for each metric.

Under the same experimental conditions, the same number of attention modules were integrated at the same position in the baseline model. The SE module significantly improved recall, reaching 92.9%, but precision decreased by 1.6% compared to Model 3, resulting in an overall lower mAP@50. The CBAM, ELA, and EMA modules did not provide performance gains. In fact, CBAM increased the parameter count while causing a decrease in mAP@50 to 96.3%, indicating poor parameter utilization. Although SimAM maintained the original parameter size, both precision and mAP@50 showed slight declines. In contrast, the MCDA module demonstrated the best overall performance, increasing both precision and recall to 95.2% and 91.9%, respectively, with mAP@50 and mAP@50–95 reaching the highest values of 97.4% and 78.9%, respectively. Despite a slight increase in the model parameter count to 3.743M after the introduction of MCDA, the significant improvement in detection accuracy justifies this increase in computational cost. Therefore, MCDA was selected as the core attention module in this study to achieve the best detection results.

### 4.7 Experimental results on the effects of inserting attention modules at different positions

To determine the optimal integration strategy for the MCDA attention mechanism within the network, detailed comparison experiments were conducted by inserting the MCDA module at different feature levels of the neck network (after the C2f-LS module, labeled -1 to -4). The experimental results are shown in **Table 5**.

**Table 5:** Experimental results on the effects of inserting attention modules at different positions.

Model	-1	-2	-3	-4	P/%	R/%	mAP@50/%	mAP@50-95/%	Params/M
Model 3					94.8	91.3	97.2	78.0	3.501
Model 3+MCDA				✓	93.7	91.6	96.8	77.1	3.680
Model 3+MCDA			✓	✓	94.5	91.8	**97.6**	78.1	3.729
Model 3+MCDA		✓	✓	✓	**95.2**	**91.9**	97.4	**78.9**	3.743
Model 3+MCDA	✓	✓	✓	✓	94.5	92.9	97.5	78.3	3.791

* Bold values represent the optimal results for each metric.

The experimental results show that the insertion of the MCDA module at mid-to-deep layers (-2, -3, -4) significantly improved detection performance, with precision, recall, mAP@50, and mAP@50–95 reaching 95.2%, 91.9%, 97.4%, and 78.9%, respectively. These values represent improvements of 0.4%, 0.6%, 0.2%, and 0.9% compared to the baseline model. However, when the MCDA module was inserted at all positions (-1 to -4), the model’s parameter count increased to a maximum of 3.791M, yet precision and mAP@50–95 decreased to 94.5% and 78.3%, respectively. This performance drop may be attributed to the fact that shallow layers primarily extract texture and edge details, and the premature introduction of attention mechanisms may lead to compression of spatial dimensions or excessive filtering of lower-level features, resulting in the loss of critical semantic information. Furthermore, inserting the module only at the deepest layer (-4) did not yield the expected performance gain; precision even dropped to 93.7%, lower than the baseline model, suggesting that attention enhancement at a single scale is insufficient to capture the diverse features of tomato fruits in complex backgrounds. In conclusion, integrating the MCDA module at multiple mid-to-deep layers not only maximizes feature extraction capabilities but also achieves the best balance between parameter count and detection accuracy.

### 4.8 Comparison experiments with other mainstream models

To evaluate the performance of the proposed YOLO-FLBM model in tomato ripeness recognition, comprehensive comparative experiments were conducted on a custom-built dataset against various mainstream models, including RT-DETR-R18, DEIM, YOLOv5s, YOLOv8s, YOLO11s, YOLO12s, YOLO13s, and YOLO26s. Furthermore, to benchmark our approach against state-of-the-art modifications specifically optimized for agricultural detection scenarios, the PC-YOLO model was introduced as an additional baseline. The experimental results are summarized in **Table 6**.

**Table 6:** Comparison experiments with other mainstream models.

Model	P/%	R/%	mAP@50/%	mAP@50-95/%	Params/M	Size/MB
RTDETR-R18	81.0	77.8	81.3	54.1	19.875	40.6
DEIM	94.3	86.8	96.2	77.8	3.720	7.8
YOLOv5s	90.3	89.1	94.7	74.8	7.822	16.0
YOLOv8s	91.5	89.4	95.5	76.5	9.828	19.9
YOLO11s	90.8	89.0	94.8	75.4	9.413	19.2
YOLO12s	91.2	88.6	95.2	75.1	9.232	18.9
YOLO13s	89.9	89.4	95.2	75.7	9.002	18.6
YOLO26s	89.1	87.2	93.9	72.6	9.466	20.3
PC-YOLO11s	90.9	91.0	96.1	77.3	8.104	16.8
YOLO-FLBM	**95.2**	**91.9**	**97.4**	**78.9**	**3.743**	**7.8**

* Bold values represent the optimal results for each metric.

The comparison results in **Table 6** clearly demonstrate that the YOLO-FLBM model outperforms the other algorithms in terms of precision, recall, and mAP@50, achieving 95.2%, 91.9%, and 97.4%, respectively. Specifically, compared to the original YOLOv8s model, YOLO-FLBM shows improvements of 3.7, 2.5, and 1.9 percentage points, highlighting the effectiveness of the proposed improvements. Regarding model complexity, YOLO-FLBM has only 3.743M parameters, a reduction of 61.91% from YOLOv8s (9.828M). The model size is 7.8 MB. These results indicate that the YOLO-FLBM model shows improvements across all metrics, with a significant increase in performance. The YOLO-FLBM model demonstrates superior comprehensive performance compared to existing cutting-edge detectors, including transformer-based architectures. By offering a lightweight design while maintaining high detection performance, it serves as a valuable reference for the deployment of vision systems in tomato harvesting robots.

### 4.9 Model visualization results

To evaluate the detection performance of YOLO-FLBM in practical greenhouse scenes, YOLOv8s and YOLO-FLBM were applied to representative tomato images collected under different environmental conditions. As shown in **Figure 9A**, the selected examples cover typical challenges in greenhouse tomato detection, including leaf occlusion, branch occlusion, strong illumination, backlighting, and fruit overlap. Compared with YOLOv8s, YOLO-FLBM produced more complete detections for partially occluded fruits and showed higher confidence scores for correctly detected targets. In scenes with strong illumination and backlighting, YOLO-FLBM also reduced false detections caused by complex background interference. These visual results indicate that the proposed model can better preserve target localization and classification performance under challenging greenhouse conditions.

To further support the qualitative visualization results, YOLOv8s and YOLO-FLBM were independently trained and evaluated three times using different random seeds under the same experimental settings. The quantitative results are presented as mean ± standard deviation in **Figure 9B**. YOLO-FLBM achieved 95.30 ± 0.26% precision, 91.93 ± 0.35% recall, 97.47 ± 0.31% mAP@50, and 78.90 ± 0.40% mAP@50-95, which were consistently higher than those of YOLOv8s (91.40 ± 0.17%, 89.63 ± 0.32%, 95.47 ± 0.15%, and 76.50 ± 0.00%, respectively). Statistical significance was analyzed using Student’s t-test at p < 0.05, and different lowercase letters indicate significant differences between the two models. These results confirm the statistical reliability of the performance gains achieved by YOLO-FLBM.

**Figure 10:**
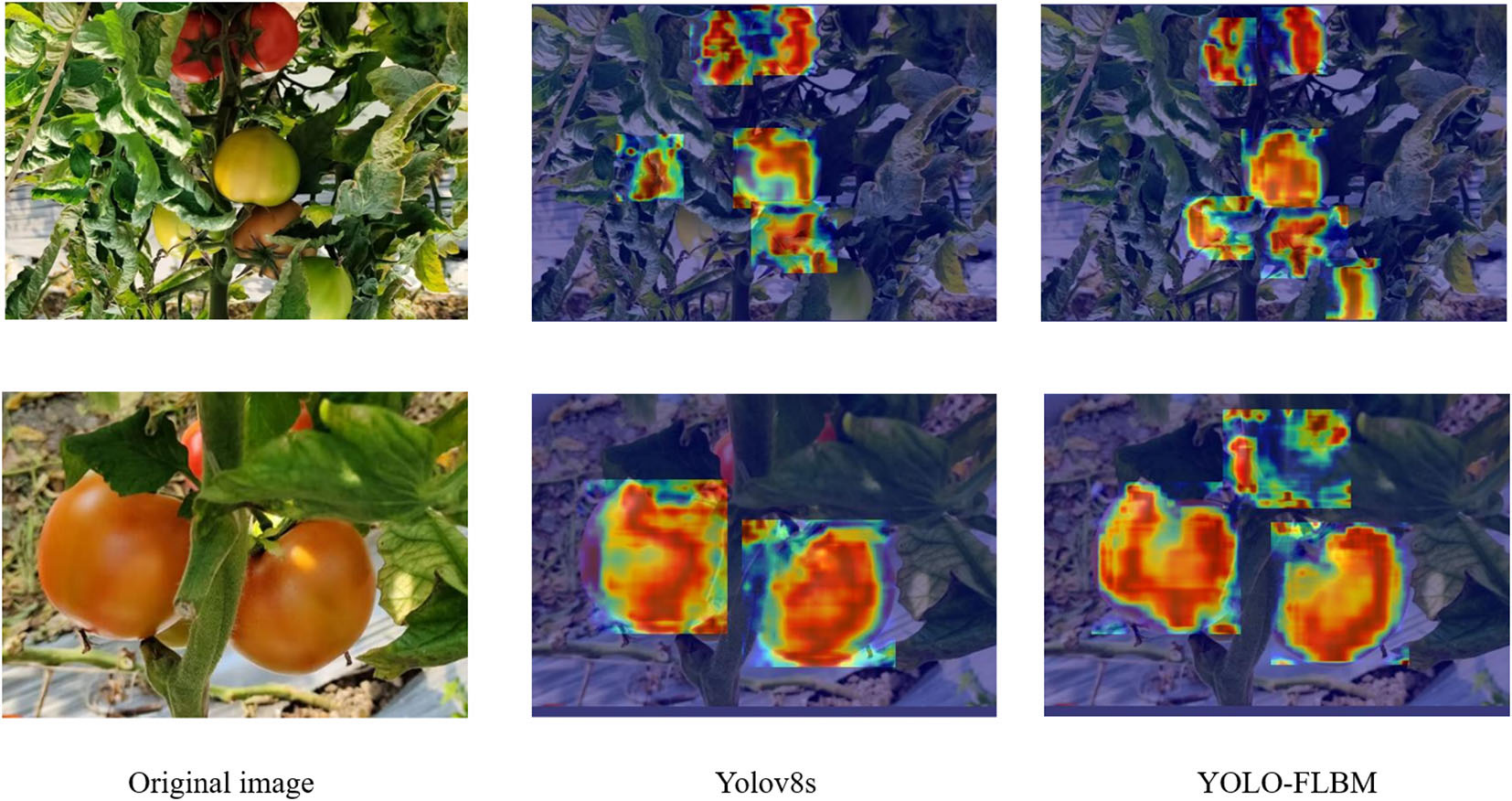
Visualization and analysis of Grad-CAM.

To analyze the contribution of the Multi-Scale Coordinate Dynamic Attention (MCDA) module, Grad-CAM was employed to visualize the model’s decision-making. **Figure 10** compares heatmaps of the baseline and YOLO-FLBM in complex greenhouse environments. In scenarios with foliage occlusion and fruit overlapping, the baseline model exhibits limitations: its heat distribution is diffuse, with attention often shifting toward background stems and leaves, indicating insufficient core feature extraction. In contrast, YOLO-FLBM demonstrates more concentrated attention, accurately anchoring tomato regions. Even under physical occlusion or overlapping, the heatmaps effectively cover visible fruit contours and respond distinctly to overlapping areas. This improvement stems from MCDA’s ability to capture spatial dependencies via multi-scale coordinate perception, enabling the extraction of key semantic features despite interference. These visualizations provide qualitative evidence that MCDA suppresses background noise and enhances feature capture for occluded targets, thereby validating its role in improving detection robustness.

## 5 Model deployment

Driven by the stringent requirements of intelligent agricultural applications, this study aimed to develop a tomato ripeness detection model that harmonizes lightweight architecture with high precision under complex greenhouse conditions. Furthermore, to validate its feasibility for edge deployment, the trained YOLO-FLBM model was converted into the RKNN format to facilitate hardware-accelerated inference, and subsequently deployed on an RK3588 development board for rigorous validation.

For the hardware deployment phase, the RK3588 development board was selected as the edge computing platform, owing to its robust hardware configuration that fully satisfies the stringent technical requirements for real-time performance and system stability inherent in tomato ripeness detection tasks. The RK3588 features an octa-core heterogeneous CPU architecture, comprising four high-performance Cortex-A76 cores and four energy-efficient Cortex-A55 cores, with a maximum clock frequency of 2.4 GHz. A critical advantage of this architecture is the integrated Neural Processing Unit (NPU), which delivers a peak computing power of 6 TOPS, providing efficient and stable hardware acceleration for the edge inference of lightweight object detection models. Regarding multimedia processing, the platform supports 8K@60 fps hardware-level video decoding and incorporates a high-performance Image Signal Processor (ISP), ensuring low-latency throughput and efficient processing under high-speed image input scenarios. To acquire high-quality *in-situ* tomato images that meet precision requirements, the system utilizes the MCIMX415 camera module (Zepin Atom, Dongguan, China) for high-resolution field image capture, alongside a 5.5-inch 1080p MIPI display configured for real-time monitoring.

**Figure 11:**
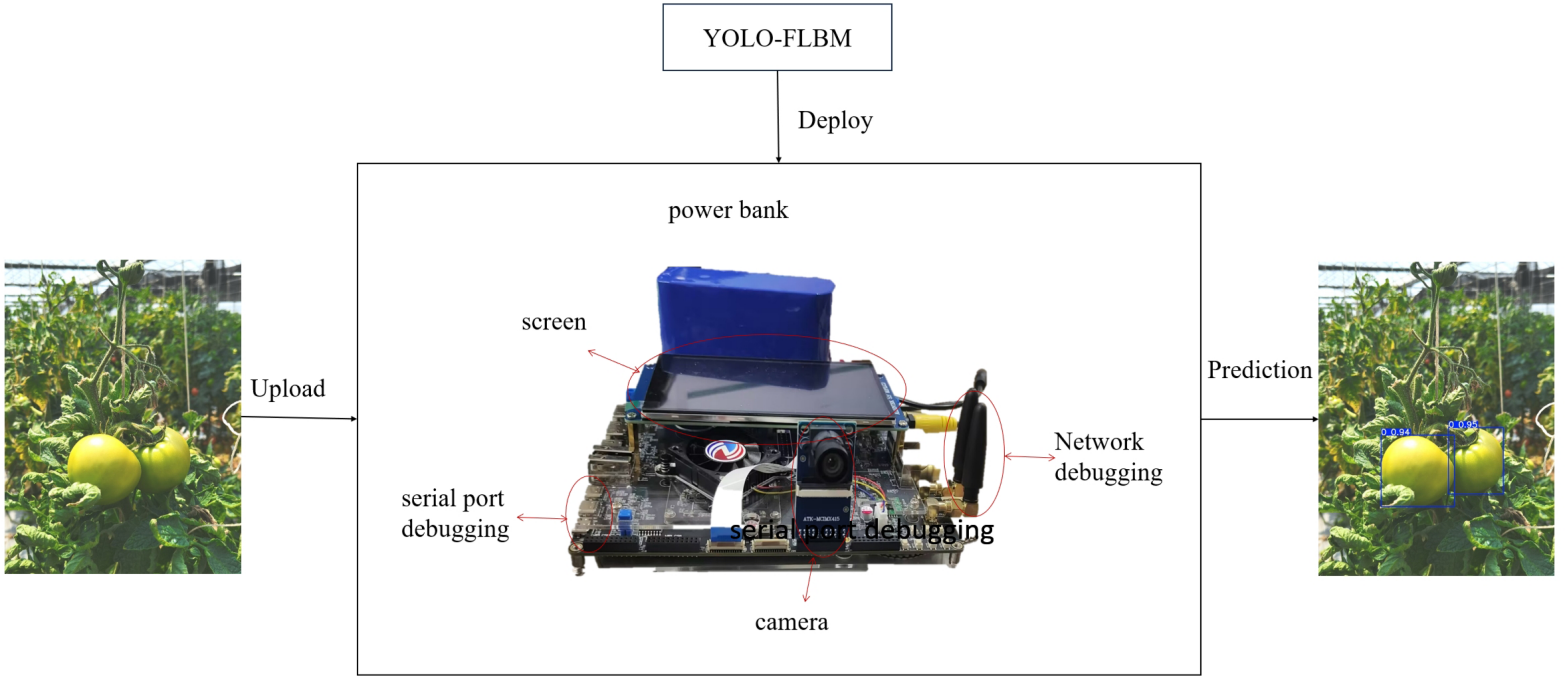
The deployed device and its detection result.

Leveraging the robust computational capabilities of the RK3588 platform, this study successfully achieved stable and real-time tomato ripeness detection within a simulated agricultural environment, thereby empirically validating the practicality and scalability of the proposed model on embedded systems. To rigorously evaluate system stability and real-time performance prior to in-field implementation, deployment experiments were conducted under controlled laboratory conditions utilizing field-acquired imagery. Performance benchmarks indicated that on a workstation equipped with an NVIDIA RTX 3080Ti GPU, the model exhibited exceptional throughput, achieving a processing speed of 408.74 FPS at an input resolution of 640 × 640 pixels. When deployed to the embedded RK3588 edge platform at the same resolution, the model maintained a high frame rate of 42.60 FPS with an average inference latency of approximately 23.47 ms per frame. Furthermore, throughout the deployment process, memory consumption remained well within the platform’s onboard resource limits, ensuring sustained and stable operation. The experimental setup and detection results under these controlled conditions are illustrated in **Figure 11**.

The proposed system reliably identifies harvestable targets and determines their spatial coordinates in 3D space. These perceptual outputs are structured for direct integration into robotic frameworks, facilitating high-performance motion planning and manipulator alignment. Consequently, this detection module functions as the foundational component of the perception layer, providing the requisite intelligence for autonomous tomato harvesting operations.

## 6 Discussion

The YOLO-FLBM tomato ripeness detection model proposed in this study maintains high detection accuracy even in complex greenhouse backgrounds. By integrating the FasterNet backbone, the LB Neck (comprising C2f-LS and BiFPN), and the Multi-scale Coordinate Dynamic Attention (MCDA) mechanism into the YOLOv8s architecture, the model’s feature extraction capability for multi-scale and occluded targets is significantly enhanced. The YOLO-FLBM model offers critical advantages for practical greenhouse management and intelligent harvesting. First, it demonstrates strong robustness in complex environments, effectively coping with foliage occlusion, fruit overlapping, and lighting variations, thereby ensuring the accuracy and stability of ripeness recognition. Second, its high inference efficiency and lightweight design enable real-time detection on resource-constrained equipment, such as harvesting robots and edge devices. Finally, the improvements in precision and recall reduce missed and false detections, minimizing operational errors and enhancing harvesting efficiency.

Despite the promising results, several limitations of this study must be acknowledged to provide a balanced view of its practical value.

First, regarding the granularity of ripeness classification, the current model categorizes tomatoes into only three stages: unripe, semi-ripe, and ripe. While this ternary classification is sufficient for basic harvesting decisions, it simplifies the continuous biological process of fruit ripening and may not fully meet the strict standards of commercial logistics, which often require finer gradation (e.g., the six-stage classification specified in the Chinese agricultural industry standard NY/T 940-2006) to optimize shelf-life management. Future work will focus on expanding the dataset to include fine-grained maturity indices, enabling the robot to execute more sophisticated selective harvesting strategies.

Second, although this study further assessed the stability of the performance gains through three independent runs with different random seeds and Student’s t-test, the statistical validation was mainly conducted for the comparison between YOLOv8s and the proposed YOLO-FLBM model on the current tomato ripeness dataset. Therefore, the robustness of the observed improvements still needs to be further examined under broader experimental settings, such as larger-scale datasets, more diverse greenhouse environments, and additional independent trials. Future work will extend the evaluation to multiple data sources and more complex deployment scenarios to further verify the generalization ability and stability of the proposed model.

Third, regarding practical deployment, although this study has successfully validated the model’s real-time inference capabilities on the RK3588 embedded platform, these evaluations were primarily conducted in a static laboratory setting. The detection module has not yet been fully integrated into the closed-loop control system of a physical robotic harvester for dynamic field operations. Consequently, potential challenges specific to kinematic environments—such as motion blur induced by manipulator velocity, mechanical vibrations, and end-effector coordination latency—remain to be quantified. To address this, our next phase will focus on mounting the RK3588-based vision system onto an autonomous harvesting robot to rigorously validate its robustness under dynamic, real-world operational conditions.

Finally, it is important to note that the development and evaluation of the proposed method were confined specifically to tomato ripeness detection within greenhouse environments. Although the growth characteristics of other solanaceous crops, such as peppers or eggplants, are known to manifest through similar visual features, further investigation is required to rigorously assess the transferability of the YOLO-FLBM model to these crops. Similarly, extending the exploration of this method to open-field cultivation systems, which are subject to more uncontrolled and variable lighting conditions compared to greenhouses, would be of significant value in establishing the broader utility and robustness of the proposed approach.

## 7 Conclusion

To effectively address the dual challenges of complex environmental interference (such as leaf occlusion and fruit overlap) and limited computational resources for real-time tomato ripeness detection, this study first constructed a tomato ripeness dataset for complex greenhouse environments. The collected images contain typical greenhouse disturbances, including dense fruit distribution, fruit occlusion, background interference, and natural illumination variations. However, the dataset was not explicitly annotated according to illumination levels; therefore, this study does not separately evaluate model performance under low-light, normal-light, and high-light conditions. Based on this dataset, we propose an improved, high-performance lightweight detection model, YOLO-FLBM. The model reconstructs the original backbone network using an optimized FasterNet architecture, constructs a novel LB Neck by implementing a dual-optimization strategy with C2f-LS and BiFPN modules, and incorporates the proposed Multi-Scale Coordinate Dynamic Attention (MCDA) mechanism for further optimization. The YOLO-FLBM model achieved a precision of 95.2%, recall of 91.9%, and mAP@50 of 97.4%, with a parameter count of only 3.743M. The results demonstrate that YOLO-FLBM significantly outperforms the baseline YOLOv8s model, reducing parameter count by 61.9% while improving detection mean Average Precision (mAP@50) by 1.9% and recall by 2.5%. These findings confirm that the YOLO-FLBM model not only maintains high detection accuracy for tomatoes in complex greenhouse environments but also retains high compactness, making it suitable for resource-constrained devices.

Future research will focus on further optimizing the YOLO-FLBM architecture to expand its applicability. We plan to augment the dataset with more diverse images that capture various ripeness stages and lighting conditions, thereby enhancing the model’s generalization ability. In particular, datasets with explicit illumination-level annotations will be constructed to systematically evaluate the model’s robustness under diverse lighting environments. The ultimate goal is to deploy this system on operational tomato harvesting robots, providing a reliable and efficient visual perception solution for smart precision agriculture.

## Data Availability

The original contributions presented in the study are included in the article/supplementary material. Further inquiries can be directed to the corresponding author.
